# Correction: Dong, W.; Song, Y. The Significance of Flavonoids in the Process of Biological Nitrogen Fixation. *Int. J. Mol. Sci.* 2020, *21*, 5926

**DOI:** 10.3390/ijms25084144

**Published:** 2024-04-09

**Authors:** Wei Dong, Yuguang Song

**Affiliations:** School of Life Science, Qufu Normal University, Qufu 273165, China; dwei@qfnu.edu.cn

In the original publication [1], the author’s first and last names in reference [14] were reversed. Reference [14] in the original publication [1] and reference [66] were not cited. These citations have now been inserted in the captions of Figure 1 and Figure 5 and should read as follows:

66. Ng, J.L.; Perrine-Walker, F.; Wasson, A.P.; Mathesius, U. The Control of Auxin Transport in Parasitic and Symbiotic Root-Microbe Interactions. *Plants* **2015**, *4*, 606–643.

With this correction, the order of some references has been adjusted accordingly. The authors state that the scientific conclusions are unaffected. This correction was approved by the Academic Editor. The original publication has also been updated.

## Figures and Tables

**Figure 1:**
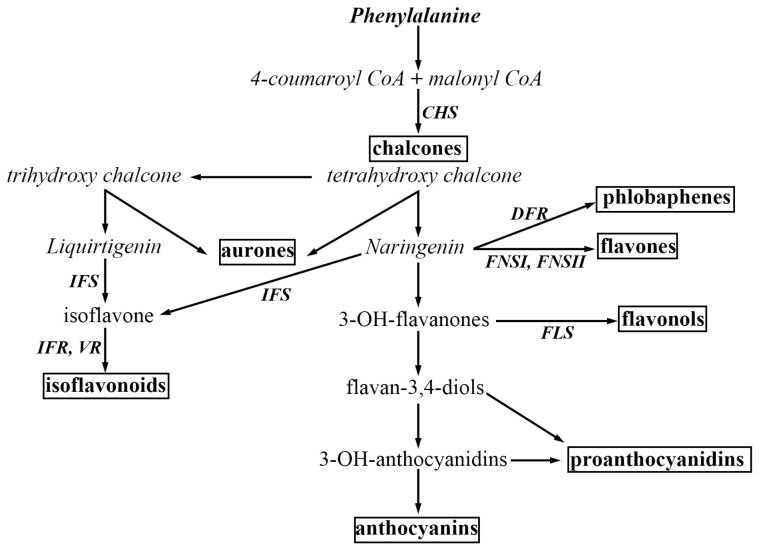
Major branches of the flavonoid biosynthesis pathway. Some of the critical enzymes are abbreviated as follows: CHS, chalcone synthase; DFR, dihydroflavonol 4-reductase; FSI/II, flavone synthase *I*/*II*; FLS, flavonol synthase; IFS, isoflavone synthase; IFR, isoflavone reductase; LCR, leucoanthocyanidin reductase; VR, vestitone reductase. Major classes of end-products are emphasized in boxes. This figure was adapted from Ref. [14].

**Figure 5:**
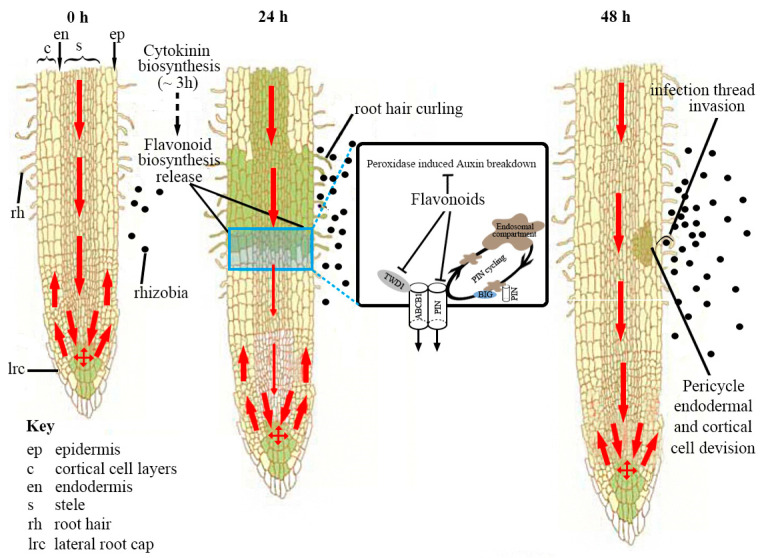
A schematic model of the regulation of auxin transport during nodulation in *Medicago truncatula*. Before rhizobia infection, auxin is transported in the acropetal direction towards the root tip. Auxin is also transported in the basipetal direction (from root tip to elongation zone) in the outer layer(s). Within 3 h after symbiosis induction (lipochitooligosaccharide treatment), cytokinin biosynthesis is upregulated in the *M. truncatula* roots [61]. Cytokinin perception at the inner cortex induces/releases certain flavonoids, which act as inhibitors of acropetal auxin transport at the inner cortical, endodermal and/or pericycle directly underlying the rhizobia infection site [62]. Flavonoids are auxin transport inhibitors that are thought to disrupt the complex between ABCB1 (ATP-Binding Cassette Subfamily B 1) and TWD1 (TWISTED DWARF1) [63,64], affecting transport, and by binding BIG, a protein required for PIN cycling [65]. The reduction in acropetal auxin transport increases the auxin concentration at the rhizobia infection site, the location of a future nodule primordium. An increase in basipetal auxin transport could also contribute to an increased auxin pool at the nodulation site [62]. Pericycle, endodermal and cortical cell divisions are activated within 48 h. The red arrow shows the polar auxin transport, and the arrow thickness is proportional to the auxin transport capacity. The green color shows the auxin gradient, and the darker color denotes a higher auxin content. This figure was adapted from Ref. [66].

## References

[1] Dong W., Song Y. (2020). The Significance of Flavonoids in the Process of Biological Nitrogen Fixation. Int. J. Mol. Sci..

